# PERCEIVED TYPES, CAUSES, AND CONSEQUENCES OF FINANCIAL EXPLOITATION: NARRATIVES FROM OLDER ADULTS

**DOI:** 10.1093/geroni/igac059.1168

**Published:** 2022-12-20

**Authors:** Annie Nguyen, Duke Han

**Affiliations:** University of Southern California, Alhambra, California, United States; University of Southern California, Alhambra, California, United States

## Abstract

We investigate the perception of financial exploitation and its causes and consequences by older adults who have firsthand experience of being exploited. Thirty-one cognitively healthy older adult participants aged 50 or older were drawn from the Finance, Cognition, and Health in Elders Study. In-depth, one-on-one interviews were conducted. Interview transcripts were analyzed using an iterative, data-driven, thematic coding scheme and emergent themes were summarized. Categories of financial exploitation included (a) investment fraud, (b) wage theft/money owed, (c) consumer fraud, (d) imposter schemes, and (e) manipulation by a trusted person. Themes emerged around perceived causes: (a) element of trust, (b) promise of financial security, (c) lack of experience or awareness, (d) decision-making, and (e) interpersonal dynamics. Perceived consequences included negative and positive impacts around (a) finances, (b) financial/consumer berhaviors, (c) relationships and trust, (d) emotional impact, and (e) future outlook. These narratives provide important insights into perceived financial exploitation and experiences.

